# GCRNN: graph convolutional recurrent neural network for compound–protein interaction prediction

**DOI:** 10.1186/s12859-022-04560-x

**Published:** 2022-01-11

**Authors:** Ermal Elbasani, Soualihou Ngnamsie Njimbouom, Tae-Jin Oh, Eung-Hee Kim, Hyun Lee, Jeong-Dong Kim

**Affiliations:** 1 Department of Computer Science and Engineering, Sun Moon University, Asan, 31460 South Korea; 2 Department of Artificial Intelligence and Software Technology, Sun Moon University, Asan, 31460 South Korea; 3 Genome-Based BioIT Convergence Institute, Sun Moon University, Asan, 31460 South Korea; 4 Department of Pharmaceutical Engineering and Biotechnology, Sun Moon University, Asan, 31460 South Korea; 5 Department of BT-Convergent Pharmaceutical Engineering, Sun Moon University, Asan, 31460 South Korea

**Keywords:** Machine learning, Drug discovery, Protein compound interaction, CNN, Bi-LSTM, Bi-GRU

## Abstract

**Background:**

Compound–protein interaction prediction is necessary to investigate health regulatory functions and promotes drug discovery. Machine learning is becoming increasingly important in bioinformatics for applications such as analyzing protein-related data to achieve successful solutions. Modeling the properties and functions of proteins is important but challenging, especially when dealing with predictions of the sequence type.

**Result:**

We propose a method to model compounds and proteins for compound–protein interaction prediction. A graph neural network is used to represent the compounds, and a convolutional layer extended with a bidirectional recurrent neural network framework, Long Short-Term Memory, and Gate Recurrent unit is used for protein sequence vectorization. The convolutional layer captures regulatory protein functions, while the recurrent layer captures long-term dependencies between protein functions, thus improving the accuracy of interaction prediction with compounds. A database of 7000 sets of annotated compound protein interaction, containing 1000 base length proteins is taken into consideration for the implementation. The results indicate that the proposed model performs effectively and can yield satisfactory accuracy regarding compound protein interaction prediction.

**Conclusion:**

The performance of GCRNN is based on the classification accordiong to a binary class of interactions between proteins and compounds The architectural design of GCRNN model comes with the integration of the Bi-Recurrent layer on top of CNN to learn dependencies of motifs on protein sequences and improve the accuracy of the predictions.

## Introduction

Compound–protein interaction (CPI) is important in the design of new compounds for the pharmaceutical industry. Proteins consist of large of small units called amino acids, which forms long chains that regulate specific functions of the human body. In humans, 20 types of amino acids are combined to form proteins. An amino acid sequence is structured into a three-dimensional complex, and its surface has a pocket that interacts with a compound through a specific combination of amino acids. In the framework of modern pharmaceutic research, the relationship between a compound and a protein can be depicted as a network, in which each node represents a compound or a protein, and an edge indicates a CPI.

Based on this paradigm, many methods based on in silico networks have been introduced to predict CPIs [1–3]. Nevertheless, these methods present limitations, such as simulating the CPI as a bipartite network while ignoring the similarities between compounds and interactions between proteins. Moreover, CPI is essential for achieving a variety of health states. In fact, compounds may be small chemical elements composed of molecules, single elements, or other combined elements that contain a variety of proteins with specific functions determined by their structure. The protein function varies according to the interaction sites that enable interaction with compounds. Thus, the interaction between molecular compounds and proteins is being actively studied for the discovery and development of safe and effective drugs. Drugs are generally low-molecular-weight compounds that regulate the biological functions of targets [4], which mostly correspond to disease-related proteins. When drugs interact with such targets, they can be used to treat the related diseases [5, 6].

The discovery of new drugs is time-consuming and costly, usually taking over 10 years in development to then conduct clinical trials for their profound study and ensure compliance with safety standards. The recording and sharing of drug information has greatly accelerated discovery and production, further facilitating the search for new interactions of drugs that can bind to more than one protein. Experimental wet laboratory experiments are available to predict interactions of known drugs, but they require considerable effort and time to set up and implement. The need for faster results has triggered the development of accurate and powerful analytical tools. Although such tools have been experimentally implemented in previous decades, current technologies and data availability have enabled the analytic process of drug development to be driven by machine learning and artificial intelligence.

Bioinformatics and data science have been combined to develop solutions based on various methods and algorithms, especially for CPI prediction [7, 8]. Conventional methods in this field use similarity-based approaches, which consider the similarity of known compound matrices with each other and across protein data. Bleakly et al. [9] proposed a model for CPI prediction with a variety of similarity information, achieving reasonable prediction performance but often demanding high computational cost, additional expertise, or three-dimensional structures of proteins.

Machine learning has been used to construct strong and sustainable drug delivery pipelines in a shorter time compared with the use of conventional methods [10]. Such pipelines allow to rapidly synthesize and analyze a small number of compounds that would help refine developed models and new designs. For drug discovery, machine learning and other technologies have enabled faster, cheaper, and more effective solutions [11].

Artificial intelligence involves various machine learning methods, with the most prominent being deep neural networks (DNNs), which provide state-of-the-art solutions in many applications, such as speech recognition and visual object recognition [12]. DNNs have also achieved excellent performance in the investigation of compounds and proteins [7, 8]. However, most available methods do not include end-to-end representation learning and consider on molecular encodings and protein phylogenetic data banks as input features that remain fixed during training. Convolutional neural networks (CNN) and recurrent neural networks (RNNs) are variants of DNNs used to classify time series and sequential data [13]. Given the long sequential nature of protein data, RNNs with long short-term memory (LSTM) layers have been proven successful. These machine learning methods can help developing high-value and cost-effective target drugs with faster transport and less harm to patients. Moreover, customized drugs can be developed to achieve the desired results faster than conventional drugs, substantially reducing the costs and time of treatments. By analyzing data of genome, proteomics, metabolomics, and clinical trials, we may fully understand the structure of a disease. Then, this knowledge may be applied to machine learning toward the development of drugs with faster and more accurate targeting.

Unlike conventional methods, a feature vector allows to automatically extract features from data without requiring expert knowledge or the three-dimensional structure of objects/proteins. Jacob et al. [14] applied tensor-product-based features to represent compound and protein families in mathematical vectors and then applied a support vector machine to predict CPIs. Jones et al. [15] used a CNN and combination graphs to find a CPI with a pairwise model, also Tsubaki et al. [16] use a similar structure for CPI prediction. Specifically in pairwise models, a CNN is used to analyze the protein structure and a graph neural network (GNN) was used for the molecular structure. Then, vectors were obtained from these branches and concatenated for the final CPI prediction.

This study contributes to the development of an end-to-end learning framework based on chemical information using a graph representation of a compound and a sequence of a protein by combining neural networks to identify the existence of CPIs. We represent compound and protein complexes as feature vectors and apply a learning algorithm to train a classifier for CPI prediction. This method, called graph convolutional recurrent neural network (GCRNN), uses protein analysis based on a CNN after a max-pooling layer followed by a bidirectional LSTM layer. The integration of recurrent layers into a CNN for protein modeling improves the representation of protein functions that dictate interactions with a compound and promote accurate results in real laboratory experiments. In addition, we integrate a recurrent layer after the max-pooling layer because protein functions follow patterns that represent specific biological arrangements, and the integration increases the detection probability and provides memory for capturing long-term dependencies [17, 18].

The remainder of this paper is organized as follows. Section 2 presents result and discussion, we draw conclusions in Sect. 3 and the proposed method is detailed in Sect. 4.

## Results and discussion

### A hybrid architecture improves the performance of prediction

After selecting the features for analyzing the data, despite factors such as data size or complexity, the performance is essential to choose the appropriate machine learning model. In addition, the selected model should deal with factors such as linearity, numbers of parameters and features of the data bank, training time, and accuracy. This work mainly measures the performance based on the classification accuracy. Also, to be noticed that the study conducted in this paper is compared with accuracy of replicated work of Tsubaki et al. [16] the model named GCNN(Graph Convolution Neural Network), with the intention improving parts of this research to convey our idea practically. The nature of data for CPI prediction is computed based on binary classes, where a class is determined by an output threshold. This work use a binary class representing the existence or absence of CPI. To accurately evaluate the model and prevent overfitting, the data were split into disjoint training (65% of the samples), validation (20% of the samples), and test (15% of the samples) sets. This work evaluate the performance using measures based on the numbers of true positives (TP), which indicate the correct classification of positive samples (i.e., CPIs), true negatives (TN), which indicate the correct classification of negative samples (i.e., no CPIs), false positives (FP), which indicate incorrect classification of positive samples, and false negatives (FN), which indicate incorrect classification of negative samples. The evaluation measures based on TP, TN, FP, and FN, and a study of performance measures for classification tasks that are used widely in learning techniques is presented in [19].

The open source genomic and protein data were retrieved from respective data repositories, for chemical structure of the compound from the PubChem database and protein sequences from the Protein Data Bank [20]. Protein and chemical data are processed in order to have a training data of compound protein interaction which is detailed from Liu et al. [21]. This work have used these data for CPI analysis, and 7000 sets of annotated data containing 1000 base length proteins have been obtained. The classes establish balanced data, and this fact demonstrate the importance of training process to have a close detection rate of each classes, raising the probability of generating a model with high accuracy. Thus, models can achieve high classification performance compared with the use of imbalanced data. We conducted experiments using various machine learning modules to evaluate different architectures.

The GNN uses a chemical input given by the simplified molecular-input line-entry system, which provides molecular encoding sequential strings. The system uses RDkit [22] to obtain graphical representations, and as an open source package include library of cheminformatics operations for compound or molecules structures.

This study use a three-layer GNN with an *r*-radius number of 2 to represent molecules as vectors. For proteins, the CNN takes the original amino acid sequence and passes through a three-layer structure with 320 convolutional kernels and a window size of 30 with random initiation based on a similar model [23]. The pooling layer has a window size of 15 and step size of 15, followed by two layers of bidirectional LSTM with 320 forward and backward neurons. The same architecture is used for the bidirectional GRU, and computations are performed over several iterations sets. The best set of hyperparameter for tuning GCRNN w\LSTM and GRU are selected to be 100 training epoch, choosing Adam optimizer, learning rate of 0.001 and decay of learning rate 0.4. The model showed high performance after tuning the hyperparameters.

This study implemented the experiments in the PyTorch [24] using a computer running the Ubuntu 18.04 operating system and equipped with an Intel i9-10,940 × processor with 256 GB memory and an NVIDIA 4xRTX2080TI graphics processor with 44 GB memory.

### Compound protein interaction prediction accuracy

**Fig. 1:**
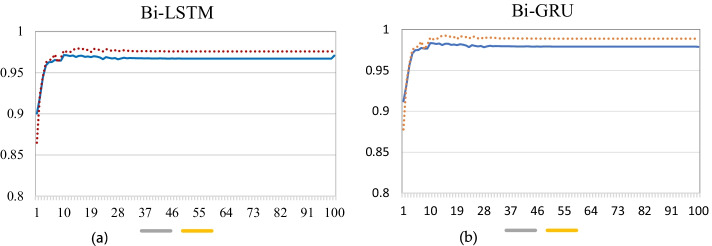
Accuracy of training and testing prediction over 100 iterations for proposed GCRNN with **a** bidirectional LSTM and **b** bidirectional GRU

CPI analysis requires wet laboratory experiments, but we only considered the data bank in this study assuming that the protein and interaction information is approved before the data were recorded. In addition, the bidirectional LSTM or bidirectional GRU after the max-pooling layer affects the CPI prediction performance. Thus, we obtained a high accuracy on the data, as shown in Fig. 1a, b for the corresponding models.

**Fig. 2:**
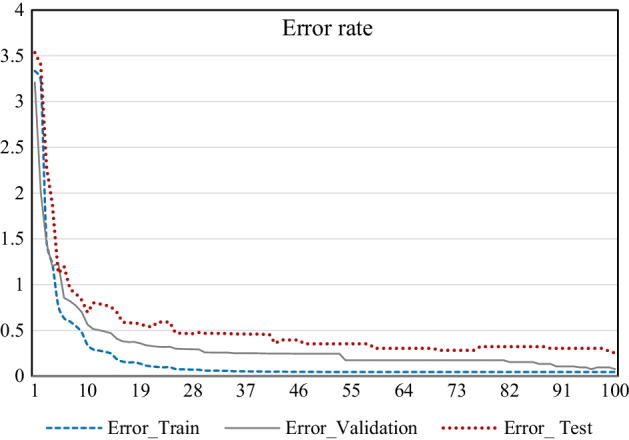
Bidirectional GRU error according to iteration

The results in Fig. 1 show that the proposed GCRNN can predict the CPIs at 98% accuracy. Further experiments related to error rate when inserting a new test set separated and the results are given for Bi directional GRU which performed better compare to Bi-LSTM and the result is visualized in the Fig. 2, showing that the training and test accuracies and the error graphs do not overfit over 100 iterations.

Compared with the GCNN, our GCRNN shows a small improvement in the overall performance, as listed in Table 1. This result suggests that the proposed GCRNN provides a more reliable prediction because protein function extraction is important for CPI. Data about proteins are available in data banks [25] and are obtained over years of research. The similarity between proteins in humans reduces the burden of data recording, and thus various calculations are facilitated by only selecting a type of protein and a type of interaction. During drug discovery, analytic results and health information are linked to recognize patterns of compounds with different proteins.

**Table 1:** Classification performance of evaluated methods for CPI prediction

Method	Precision	Recall	Accuracy	F1-measure
GCNN	0.92	0.94	0.96	0.92
GCRNN w/LSTM	0.95	0.92	0.97	0.93
GCRNN w/GRU	0.97	0.94	0.98	0.95

Visualization tools provide insights on the medical outcomes expected for patients with a high accuracy to predict effects while reducing the time and setup workload of wet laboratory experiments for producing a specific drug. With the advancement of research, data banks will become larger, increasing our ability to understand CPI patterns for healthcare, and patients will be treated with specific drugs related to their health condition.

This research is limited to the analysis process of the framework, even why several implementations are performed, a confident discussion of compound protein interaction requires wet laboratory experiments to be associates with, but this work will focus only on the database supposing that protein and interaction information is approved before when data are recorded.

## Conclusions

This work proposes GCRNN to identify CPIs using high-end machine learning methods. Also, emphasize the end-to-end representation learning of a GNN and a CNN with bidirectional LSTM/GRU to predict CPIs. Experimental results demonstrate that a relatively low-dimensional end-to-end neural network can outperform various existing methods on both balanced and imbalanced data.

This study provides new insights on CPI prediction to construct general machine learning methods in bioinformatics rather than using feature engineering. Unlike existing structure-based computational approaches, the proposed GCRNN shows high performance using only protein primary structure information instead of three-dimensional structure information. Nevertheless, a deep learning model is usually considered a black box. Consequently, it is difficult to interpret the features that the model learns for CPI prediction. Improving the prediction performance on the validation and test sets would provide a starting point for subsequent research. In future work, this study will evaluate the model learning and performance considering comparisons with the results from wet laboratory experiments.

## Methods

### GCRNN for CPI prediction

Machine learning and computational methods are enhancing data analysis on a large scale and providing faster solutions, impacting research on biology and pharmaceutics. Biological data have been collected in data banks with plenty of information about genome and proteins being available for researchers to obtain reliable results in areas such as healthcare.

**Fig. 3:**
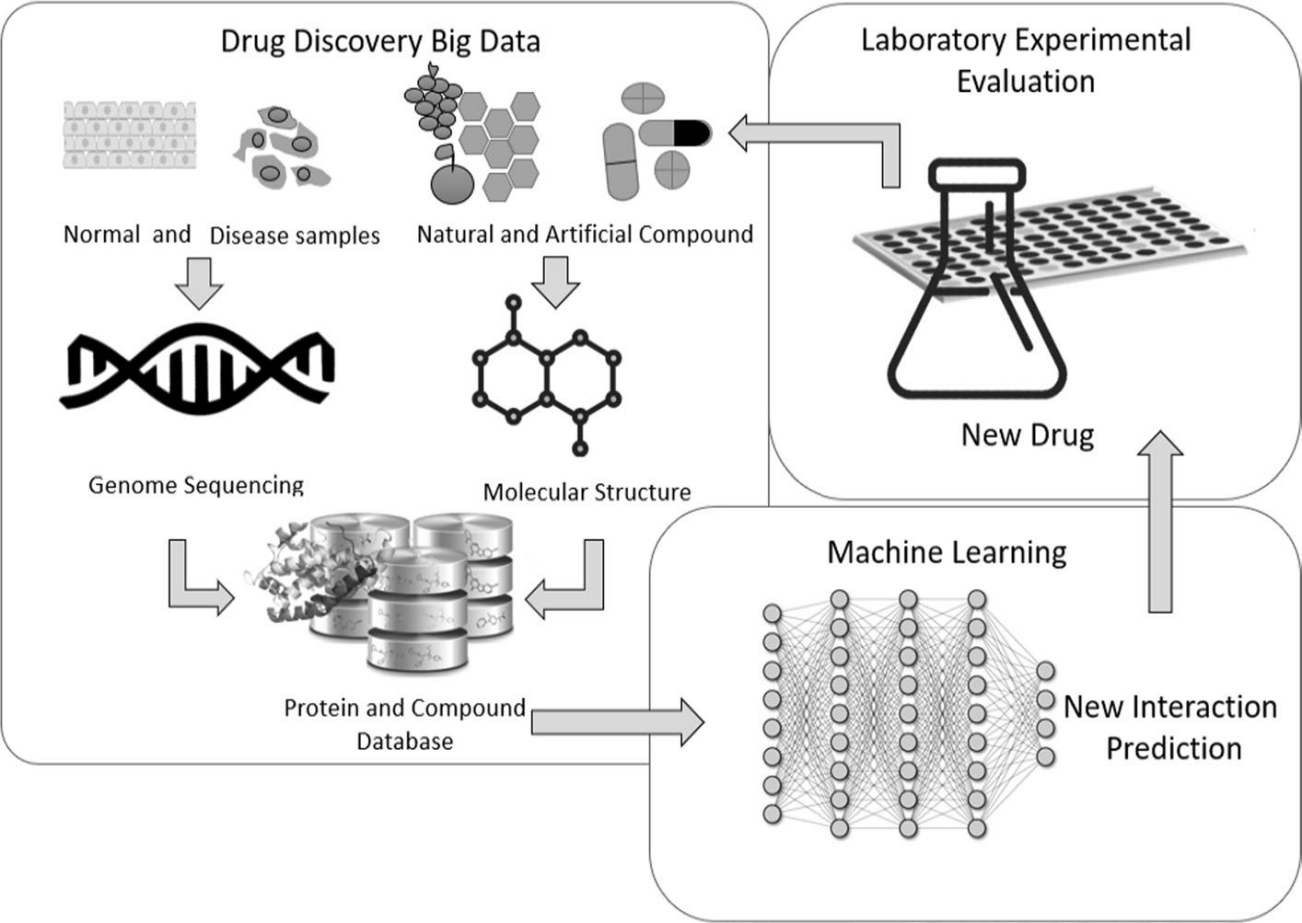
Deep learning-based drug discovery approach

This work address CPI prediction, an important aspect for drug discovery and development. Figure 3 illustrates the development of new drugs for improving health conditions based on the information of proteins and chemical structure of natural or artificial compounds.

A normal or abnormal condition carries information in the genome sequence, which can be translated into a protein sequence that interacts with a compound. The interactions can be stored continuously by using machine learning to determine the effective compound and protein for a specific disease. Then, laboratory experiments provide accurate results for clinical trials, and the resulting compound extends the dataset for new cases and developments.

**Fig. 4:**
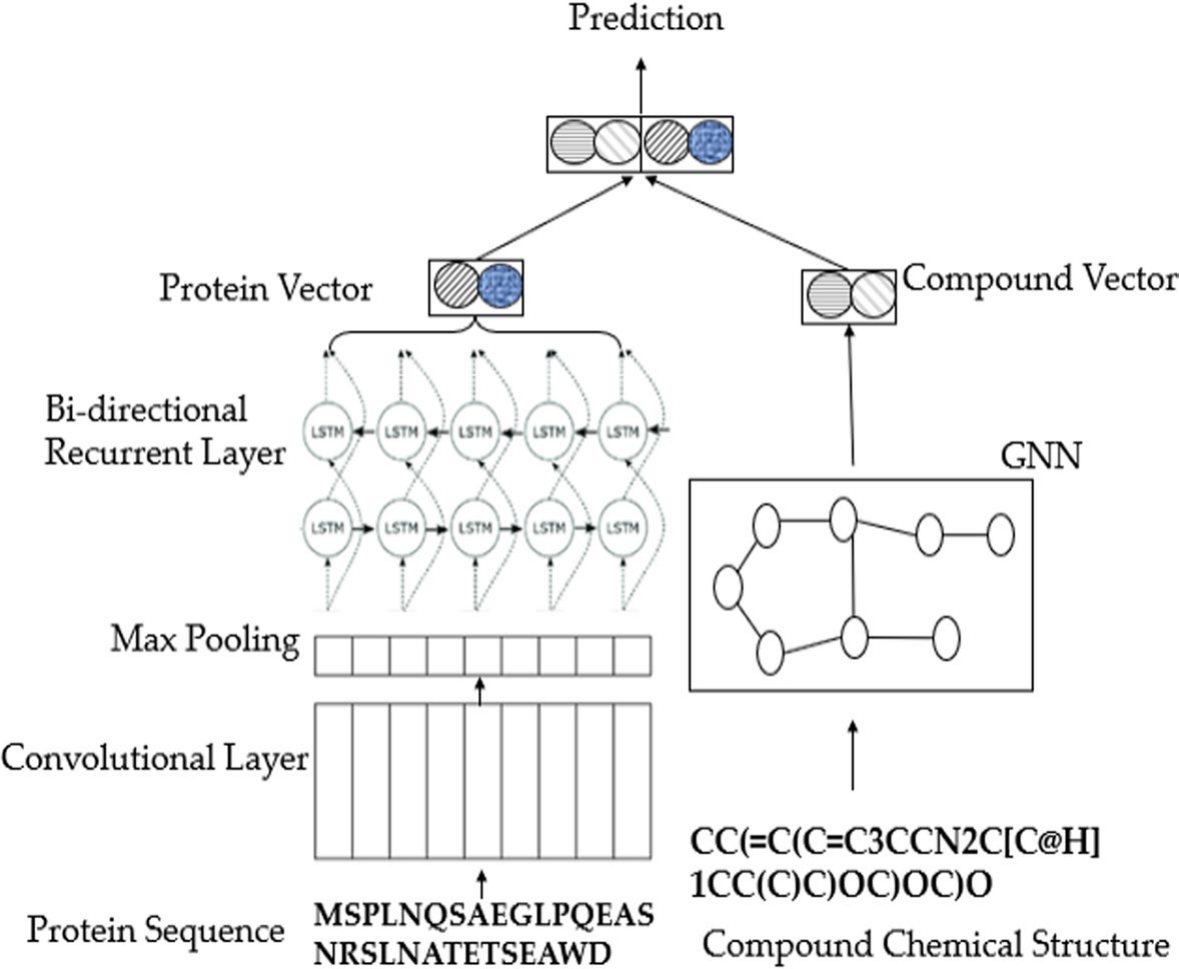
Architecture of GCRNN for CPI prediction

Deep learning techniques provide state-of-the-art performance and high accuracy for handling protein sequences and modeling molecules. Among the available models and architectures, we combine three powerful methods for CPI prediction, namely, GNN, CNN, and bidirectional RNN, as shown in Fig. 4. These methods constitute the proposed GCRNN and are detailed below.

#### GNN

The GNN can provide the low-level error vector of a molecular chart. We use the GNN to represent a molecular embedding that maps a graph into a vector through transformation and output functions. In the GNN, the transformation function updates the node values related to the neighboring nodes and edges, and the output function describes the nodes as vectors. In the graph structure, G = (N, E), where N is the set of nodes, and E is the set of edges that connect neighboring nodes. We consider undirected graph G, in which a node *n*_*i*_ ∈ N represents atom *i* of a molecule, and *e*_*ij*_ ∈ E represents the bond between atoms *n*_*i*_ and *n*_*j*_.

Considering molecules as graphs simplifies the representation by defining few types of nodes and bonds and few parameters to learn. We also adopt *r*-radius subgraphs [26] that outperform the representation learning of the number of neighboring nodes. In an *r*-radius subgraph, for graph G = (N, E), the set of all nodes within a radius *r* of node *i* are represented as *S*(*i*,*r*), and the subgraph of *r*-radius nodes *n*_*i*_ is defined as

1 $$n_{i}^{\left( r \right)} = \left( {N_{i}^{\left( r \right)} ,E_{i}^{\left( r \right)} } \right),$$

where $N_{i}^{\left( r \right)} = \left\{ {n_{j} \left| {j \in S\left( {i,r} \right)} \right.} \right\}$ and $E_{i}^{\left( r \right)} = \left\{ {e_{mn} {|}\left( {m,n} \right) \in S\left( {i,r} \right) \times S\left( {i,r - 1} \right)} \right\}$. The subgraph for the *r*-radius edges is defined as

2 $$e_{ij}^{\left( r \right)} = \left( {N_{i}^{{\left( {r - 1} \right)}} \cup N_{j}^{{\left( {r - 1} \right)}} ,E_{i}^{{\left( {r - 1} \right)}} \cup E_{j}^{{\left( {r - 1} \right)}} } \right).$$

An embedded vector is assigned for the *r*-radius node and *r*-radius edge, which are randomly initialized, and backpropagation is used for training. To update the node information with respect to its neighborhood, the transition functions in Eqs. (3) and (4) are used. At time step *t* of a given graph with random embeddings of nodes and edges, *n*(*t*) represents a node in Eq. (3) and *e*(*t*) represents an edge in Eq. (4). The updated vectors are defined as

3 $$n_{i}^{{\left( {t + 1} \right)}} = \sigma \left( {n_{i}^{\left( t \right)} + \mathop \sum \limits_{j \in S\left( i \right)} p_{ij}^{\left( t \right)} } \right),$$

where σ is the sigmoid function [27] and $$p_{ij}^{\left( t \right)} = f\left( {W_{{{\text{neighbor}}}} \left[ {\begin{array}{*{20}c} {n_{j}^{\left( t \right)} } \\ {e_{ij}^{\left( t \right)} } \\ \end{array} } \right] + b_{{{\text{neighbor}}}} } \right)$$ is a neural network with *f* being a ReLU (rectified linear unit) activation function [27] and W_neighbor_ and *b*_neighbor_ being a weight matrix and bias vector, respectively, at time step *t*. In the same iteration, the edges are updated as follows:

4 $$e_{i}^{{\left( {t + 1} \right)}} = \sigma \left( {e_{i}^{\left( t \right)} + q_{ij}^{\left( t \right)} } \right),$$

where function *q* is the neural network model for the edges and $q_{ij}^{\left( t \right)} = f\left( {W_{{{\text{side}}}} \left( {n_{i}^{\left( t \right)} + n_{j}^{\left( t \right)} } \right) + b_{{{\text{side}}}} } \right)$. The transition functions generate an updated set of nodes ${\varvec{N}} = \left\{ {{\varvec{n}}_{1}^{{\left( {\varvec{t}} \right)}} ,{\varvec{n}}_{2}^{{\left( {\varvec{t}} \right)}} ,..,{\varvec{n}}_{{\left| {\varvec{N}} \right|}}^{{\left( {\varvec{t}} \right)}} } \right\}$, where |N| is the number of nodes in the molecular graph. Finally, the molecular representation vector is given by

5 $$y_{{{\text{molecule}}}} = \frac{1}{\left| N \right|}\mathop \sum \limits_{i = 1}^{\left| N \right|} n_{i}^{\left( t \right)} .$$

#### CNN

CNNs are DNNs that also effective for analyzing protein sequences. As a CNN uses a weight-sharing strategy to capture local patterns in data, it is suitable for studying DNA (deoxyribonucleic acid) because convolution filters can determine functions of protein sequences that are short repeating patterns in DNA that may have a biological function. The proposed deep CNN is characterized by sequential interactive convolutional and pooling layers that extract features form sequence at various scales, followed by a fully connected layer that computes the whole-sequence information to extract protein features. Each CNN layer undergoes a linear transformation from the previous output. Then, it is multiplied by a weight matrix and proceeds with a nonlinear transformation. To minimize prediction errors, the weighted value matrix is learned during training. The CNN model base layer is a convolutional layer that calculates the output of a one-dimensional operation concerning a specific number of kernels (weight matrices later transformed by ReLU activation). The CNN for proteins maps sequence P into vector y with multiple filter functions. The first CNN layer is applied to proteins, where the *n*-gram (*n* = 3) technique allows to represent amino acids as words. The group of three overlapping amino acids makes a word and represents input sequence P. The convolution for input protein sequence P is defined as

6 $$convolution\left( P \right)_{ik}^{\left( t \right)} = ReLU\left( {\mathop \sum \limits_{m = 0}^{M - 1} \mathop \sum \limits_{n = 0}^{N - 1} W_{mn}^{k} P^{\left( t \right)}_{i + m,n} } \right),$$

where *i* is the index of the output position, *k* is the index of the kernels, and *W*^*k*^ is an *M* × *N* weight matrix with *M* windows and *N* input channels. Then, a max-pooling layer reduces the size of the input or hidden layers by choosing the maximally activated neuron from a convolutional layer. Accordingly, for the CNN to be independent of the length of the protein sequence, max-pooling is applied when the maximally activated neuron is selected from the convolutional layer. Consequently, the number of hidden neurons generated by the convolution filter is the same as that of filters and not affected by the length of the input. For input *Q*, pooling is defined as

7 $$pooling\left( Q \right)_{ik}^{\left( t \right)} = {\text{max}}\left( {Q_{iM,k} ,Q_{{\left( {iM + 1,k} \right)}} , \ldots ,Q_{{\left( {iM + M - 1,k} \right)}} } \right).$$

#### Bidirectional RNN

An RNN is another type of DNN. Unlike a CNN, the connections between the RNN units form a directed cycle that creates an internal state of the network to exhibit a dynamic temporal or spatial behavior. A bidirectional LSTM is a variant of the RNN that combines the outputs of two RNNs to process a sequence both from left to right and from right to left. Instead of regular hidden units, the two proposed RNNs contain LSTM layers, which are smart network units that can remember a value over an arbitrary period. A bidirectional LSTM can capture long-term dependencies and has been effective for various machine learning applications. Bidirectional gated recurrent units (GRUs) are an alternative to bidirectional LSTMs to constantly represent sequential input without using separate memory units [28]. We use LSTM and GRU to prove that adding a recurrent structure after the CNN increases performance.

Features V provided from the pooling layer form a sequence ${\varvec{x}} = \left\{ {{\varvec{x}}_{1} ,{\varvec{x}}_{2} , \ldots ,{\varvec{x}}_{{\varvec{V}}} } \right\}$, which serves as input for a two-layer bidirectional neural network. The bidirectional LSTM layers updated at step *v* depend on forward and backward processing as follows:

8 $$\begin{aligned} \vec{i}_{t} & = \sigma \left( {\vec{W}_{i} \left[ {x_{t} ,\vec{h}_{t - 1} } \right] + \vec{b}_{i} } \right), \\ \vec{f}_{t} & = \sigma \left( {\vec{W}_{f} \left[ {x_{t} ,\vec{h}_{t - 1} } \right] + \vec{b}_{f} } \right), \\ \vec{o}_{t} & = \sigma \left( {\vec{W}_{o} \left[ {x_{t} ,\vec{h}_{t - 1} } \right] + \vec{b}_{o} } \right), \\ \vec{g}_{t} & = tanh\left( {\vec{W}_{c} \left[ {x_{t} ,\vec{h}_{t - 1} } \right] + \vec{b}_{c} } \right), \\ \vec{c}_{t} & = \vec{f}_{t} \odot \vec{c}_{t - 1} + \vec{i}_{t} \odot \vec{g}_{t} , \\ \vec{h}_{t} & = \vec{o}_{t} tanh\left( {{ }\vec{c}_{t} } \right) \\ H & = \vec{W}_{h} \vec{h}_{t} + \mathop{W}\limits^{\leftarrow} _{h} \mathop{h}\limits^{\leftarrow} _{t} . \\ \end{aligned}$$

At time *t*, → and ← indicate the calculation direction, *i* is the input gate, *f* is the forget gate, *o* is the modulate gate, *h* is the hidden state at time *t*, *W*_*i*_, *W*_*F*_, *W*_*o*_, and *W*_*c*_ are weight matrices for their corresponding gates, and $\odot$ denotes the elementwise multiplication. The equivalent bidirectional GRU is defined as follows:

9 $$\begin{aligned} \vec{z}_{t} & = \sigma \left( {\vec{W}_{z} \left[ {x_{t} ,\vec{h}_{t - 1} } \right] + \vec{b}_{z} } \right), \\ \vec{r}_{t} & = \sigma \left( {\vec{W}_{r} \left[ {x_{t} ,\vec{h}_{t - 1} } \right] + \vec{b}_{r} } \right), \\ \overrightarrow {{\tilde{h}}}_{t} & = tanh\left( {\vec{W}_{h} \left[ {x_{t} ,r_{t} \odot \vec{h}_{t - 1} } \right]} \right), \\ \vec{h}_{t} & = \left( {1 - z_{t} } \right) \odot \vec{h}_{t - 1} + \vec{g}_{t} \odot \overrightarrow {{\tilde{h}}}_{t} , \\ \end{aligned}$$

where *r* is the reset gate and *z* is the update gate. The LSTM/GRU encodes $\vec{\user2{h}}_{{\varvec{t}}} \user2{ }$ along the left direction of the embedded protein at position *t*. As both the left and right directions are important for the global structure of proteins, we use a bidirectional LSTM (or bidirectional GRU). The bidirectional layers encode each position into leftward and rightward representations. H is the output, which is the sum of the results along both directions:

11 $$H = \vec{W}_{h} \vec{h}_{t} +\overleftarrow {W} _{h}\overleftarrow {W} _{t},$$

where H is a set of hidden vectors $H = \left\{ { h_{1}^{{\prime}\;(t)} , h_{2}^{{\prime}\;(t)} , \ldots , h_{\left| V \right|}^{{\prime}\;(t)}} \right\}$ obtained from the bidirectional LSTM/bidirectional GRU output. The protein vector representation is given by

12 $$y_{{{\text{protein}}}} = \frac{1}{\left| V \right|}\mathop \sum \limits_{i = 1}^{\left| V \right|} b_{i}^{\left( t \right)}$$

The vectors are concatenated to obtain output vector ${\text{Out}} = W_{{{\text{out}}}} \left[ {y_{{{\text{molecule}}}} ;y_{{{\text{protein}}}} } \right] + b_{{{\text{out}}}}$, which is the input of a classifier, where ${\varvec{W}}_{{{\mathbf{out}}}}$ is a weight matrix and ${\varvec{b}}_{{{\varvec{out}}}}$ is a bias vector. Finally, softmax activation is added to vector **Out**[${\varvec{y}}_{0} ,{\varvec{y}}_{1}$] to predict a binary label that represents the existence or not of a CPI.

## Data Availability

Contact the corresponding author and the dataset is same described also in https://doi.org/10.1093/bioinformatics/btv256.

## Abbreviations

GCRNN: Graph convolution recurrent neural network
CNN: Convolution neural network
LSTM: Long Short-Term Memory
Bi-LSTM: Bi-directional LSTM
GRU: Gated recurrent units
Bi-GRU: Bi-directional GRU
CPI: Compound-protein interaction
DNN: Deep neural network
RNN: Recurrent neural network
GNN: Graph neural network
DNA: Deoxyribonucleic acid
TP: True positive
TN: True negative
FP: False positive
FN: False negative
ReLU: Rectified Linear Unit
